# Neuroprotective effects of Ershiwuwei Shanhu pills on APP/PS1 mice through antioxidant enhancement, anti-apoptosis, and MAPK pathway regulation

**DOI:** 10.1515/tnsci-2025-0396

**Published:** 2026-06-22

**Authors:** Ning Li, Xiaoyuan Peng, Wei Xiong, Ce Yang, Wenxiang Wang

**Affiliations:** Chongqing Three Gorges Medical College, Chongqing, China; Chongqing Key Laboratory of Development and Utilization of Genuine Medicinal Materials in Three Gorges Reservoir Area, Chongqing, China

**Keywords:** Tibetan medicine, memory deficits, neuronal injury, oxidative stress, signal transduction

## Abstract

**Objectives:**

Alzheimer’s disease (AD) involves cognitive impairment, neuronal degeneration, oxidative imbalance, and abnormal MAPK signaling. This study investigated the protective effects of Ershiwuwei Shanhu Pills (ESP) on cognition, oxidative stress, neuronal apoptosis, and MAPK pathway regulation in APP/PS1 mice.

**Methods:**

Sixty mice were used, including 50 APP/PS1 transgenic mice randomly assigned to five groups: untreated AD model, donepezil (0.5 mg/kg), and low- (100 mg/kg), medium- (200 mg/kg), or high-dose (400 mg/kg) ESP. Ten wild-type C57BL/6J mice served as normal controls. All treatments were administered orally for 60 days. Cognitive performance was assessed by the Morris water maze. Hippocampal pathology and apoptosis were evaluated by histology and TUNEL staining, while oxidative stress markers, AD-related proteins, and MAPK phosphorylation were measured via ELISA and Western blot.

**Results:**

ESP treatment improved learning and memory performance, reduced hippocampal neuronal damage, and decreased neuronal apoptosis. Antioxidant enzyme activities (SOD, CAT, GSH, GSH-PX) increased, whereas MDA and GSSG levels decreased. Circulating Aβ1-40, Aβ1-42, TAU181, and γ-secretase levels were reduced. ESP also downregulated phosphorylation of JNK, ERK, and p38. The medium-dose group showed therapeutic effects comparable to donepezil.

**Conclusions:**

ESP exerts neuroprotective effects in APP/PS1 mice by alleviating oxidative stress, inhibiting neuronal apoptosis, and modulating MAPK signaling. These findings suggest ESP as a promising multi-target therapeutic strategy for AD.

## Introduction

Alzheimer’s disease (AD) is a chronically evolving neurodegenerative condition characterized by declining cognitive faculties and widespread neuronal degeneration, especially among the aging population, posing significant healthcare challenges worldwide [1], [2], [3]. The disease is pathologically defined by amyloid-beta (Aβ) plaque accumulation, neurofibrillary tangles, and widespread neuronal degeneration [4], [5], [6]. The hippocampus, a critical region for learning and memory, is among the earliest and most severely affected brain areas in AD, with its dysfunction correlating strongly with cognitive impairment [7], 8]. Emerging evidence points to oxidative stress (OS) and dysregulated MAPK signaling as major contributors to AD pathogenesis [9], 10]. OS, resulting from excessive reactive oxygen species (ROS), disrupts redox homeostasis, damages macromolecules, and promotes neuronal death [9], 11], 12]. AD models typically exhibit decreased activities of antioxidant enzymes, including superoxide dismutase (SOD), catalase (CAT), and glutathione peroxidase (GSH-PX), alongside elevated levels of malondialdehyde (MDA) and oxidized glutathione (GSSG), reflecting oxidative damage associated with Aβ accumulation [13], [14], [15].

The MAPK signaling pathway, encompassing ERK, JNK, and p38 sub-pathways, regulates cellular stress responses, survival, and differentiation. In AD, aberrant activation of these cascades enhances Aβ production and tau hyperphosphorylation, thereby accelerating neurodegeneration [16], [17], [18]. Recent studies support the therapeutic potential of MAPK inhibition; for instance, selective suppression of p38α/β MAPK has been documented to improve both histopathology and cognitive function in AD mouse models [19], 20].

Ershiwuwei Shanhu Pills (ESP), a traditional Tibetan multi-herb formulation that includes coral, have historically been used to manage neurological and hematological disorders such as epilepsy and leukopenia [21]. Clinical data indicate that ESP, particularly when combined with levetiracetam, enhances seizure control and modulates inflammatory cytokines, including IL-2, IL-6, TNF-α, and CRP [21]. Furthermore, ESP has been reported to facilitate cerebral microcirculation and vasodilation, potentially supporting brain perfusion under pathological conditions [22]. Although its mechanisms remain incompletely defined, preliminary findings suggest ESP exerts anti-inflammatory, antioxidative, and neuroprotective effects [23], 24]. Nonetheless, its efficacy in neurodegenerative disorders like AD has not been systematically explored. Traditionally, Tibetan medicine emphasizes long-term administration for chronic conditions. Considering that AD is a progressive disease, a prolonged treatment window is essential to observe significant modification in pathology. In APP/PS1 mice, ages 6–8 months mark a key stage of accelerated amyloid deposition and cognitive deterioration, making a 60 day treatment window appropriate for assessing therapeutic effects.

Given the involvement of oxidative stress, apoptosis, and MAPK signaling in AD and the multi-target properties of ESP, this study investigates its neuroprotective effects in APP/PS1 mice. Through behavioral analysis, histopathological evaluation, and molecular profiling, we sought to determine whether ESP mitigates cognitive dysfunction and neuropathological alterations by modulating OS, neuronal apoptosis, and MAPK signaling. This work may provide a foundation for repositioning ESP as a potential therapeutic agent in AD and deepen our understanding of the disease’s molecular underpinnings.

## Materials and methods

### Experimental animals

Six-month-old male SPF C57BL/6J and APP/PS1 mice were purchased from Jiangsu Huachuang XinNuo Pharmaceutical Technology Co., Ltd. (Experimental Animal License No.: SCXK [Su] 2020-0009) and housed in the SPF facility of Chongqing Three Gorges Medical College under controlled temperature (24 ± 2 °C), humidity (50 ± 10 %), and a 12 h light/dark cycle, with free access to food and water. Prior to the experiment, all animals underwent a one-week acclimatization period. All experimental protocols were reviewed and approved by the Biomedical Ethics Committee of Chongqing Three Gorges Medical College (Approval No.: SYYZ-A-2212-0003).

### Drugs and reagents

ESP were obtained from Jinhe Tibetan Medicine Co., Ltd. (National Medicine Standard No.: Z63020059, Batch No.: 01230616). To ensure the reproducibility and quality consistency of the formulation, qualitative and quantitative analyses were performed based on the manufacturer’s quality analysis certificate and the Chinese Pharmacopoeia (2020 edition). Major bioactive constituents were determined by High-Performance Liquid Chromatography (HPLC) and atomic absorption spectroscopy. The primary ingredients include Coral (Coral skeleton), Pearl (Pteria martensii), Cinnabar (HgS), and Saffron (*Crocus sativus*). Quantitative analysis revealed that the content of Cinnabar (calculated as HgS) was 12.5 %, and the content of Crocin-I (C44H64O24) from Saffron was not less than 0.35 mg/g. The specific composition and content ranges of the major markers are detailed in Table 1. Donepezil (Don) hydrochloride tablets were provided by Pfizer Australia Pty Ltd (ARTG ID: 123456, Batch No.: XYZ123).

**Table 1:** Major bioactive components and concentrations in Ershiwuwei Shanhu pills (ESP).

Component	Chemical marker/active substance	Detection method	Concentration/content range
Coral (Shanhu)	Calcium carbonate (CaCO3)	Titration	>95.0 % (of skeleton weight)
Pearl (Zhenzhu)	Calcium carbonate (CaCO3)	Titration	>90.0 %
Cinnabar (Zhusha)	Mercuric sulfide (HgS)	Titration/AAS	12.0–13.0 % (w/w)
Saffron (Xihonghua)	Crocin-I (C44H64O24)	HPLC	≥0.35 mg/g
Costus root (Muxiang)	Costunolide	HPLC	≥0.80 mg/g
*Aucklandia lappa*	Dehydrocostus lactone	HPLC	≥1.20 mg/g
Licorice (Gancao)	Glycyrrhizic acid	HPLC	≥1.50 mg/g

Data represent the mean values from three independent batches analyzed according to the Chinese pharmacopoeia (2020 edition) standards. AAS, atomic absorption spectroscopy; HPLC, high-performance liquid chromatography.

### Animal grouping and drug administration

**Figure 1:**
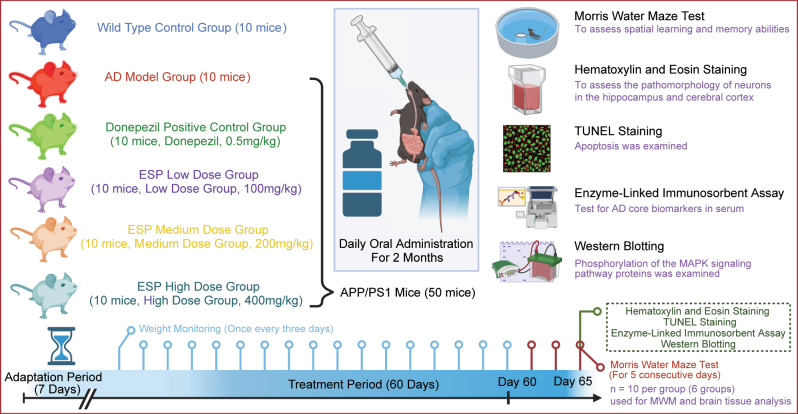
Experimental flowchart.

Fifty APP/PS1 mice were randomly divided into five groups (n=10): model (APP/PS1), Don (0.5 mg/kg), low-dose ESP (ESPL, 100 mg/kg), medium-dose ESP (ESPM, 200 mg/kg), and high-dose ESP (ESPH, 400 mg/kg). The selection of ESP dosages was based on the conversion of the standard human clinical daily dose (approximately 1 g/day) to the murine equivalent using the body surface area normalization method, which corresponds to the medium dose (200 mg/kg). The low (100 mg/kg) and high (400 mg/kg) doses were subsequently set based on our preliminary dose-response experiments to evaluate the dose-dependent neuroprotective efficacy. An additional ten wild-type C57BL/6J mice served as the normal control group (WT). All experimental groups were administered treatments via daily oral gavage for a duration of 60 consecutive days. Body weight was recorded every three days during the treatment period. At the end of the dosing period, a five-day Morris Water Maze (MWM) behavioral test was conducted, with a probe trial performed on the fifth day. Following the final test, mice were euthanized, and serum and brain tissue samples (primarily hippocampal regions) were collected for subsequent analyses (Figure 1).

### MWM test

Spatial learning and memory were assessed using the Morris water maze. Mice were trained in a circular pool (150 cm diameter, 40 cm depth; 23–25 °C) filled with opaque water, with a hidden platform (9 cm diameter, 1 cm below the surface) placed in a fixed quadrant. Training was conducted over five days with four trials per mouse per day, and escape latency was recorded. Mice that failed to find the platform within 60 s were guided to it and allowed to remain for 15 s. On day 5, a probe trial was performed after platform removal, and the time spent in the target quadrant during 60 s was measured. Behavioral data were automatically recorded using the ZS001 system (Beijing Zhongshi Technology Co., Ltd.).

### Hematoxylin and eosin (H&E) and nissl staining

After the MWM test, hippocampal tissue from three randomly selected mice in each group was used for H&E and Nissl staining [25].

Brain tissues were fixed in 4 % paraformaldehyde, dehydrated through graded ethanol, paraffin-embedded, and sectioned coronally at 5 μm using a Leica RM2235 microtome (Leica, Wetzlar, Germany). Sections were stained with hematoxylin-eosin (H&E) following standard procedures, including hematoxylin staining, differentiation, bluing, eosin counterstaining, dehydration, and xylene clearing. Images were captured using a DM1000 imaging system (Leica, Germany) coupled with an optical microscope (CX21FS1, Olympus Corporation, Japan) [26].

Nissl staining was performed using the G1036 kit (Servicebio) following sequential ethanol dehydration, rinsing with distilled water, staining, differentiation, and mounting. Images were captured using the DM1000 imaging system (Leica) and the CX21FS1 optical microscope (Olympus).

### TUNEL staining

Hippocampal neuronal apoptosis was assessed using a red fluorescent TUNEL assay kit (G1502, Servicebio). Deparaffinized 5 µm paraffin sections were fixed and permeabilized, incubated with TdT buffer at 37 °C for 10 min, and then reacted with the TdT labeling solution at 37 °C for 1 h. Nuclei were counterstained with DAPI (G1012, Servicebio) in the dark for 10 min, and anti-fade mounting medium (G1401, Servicebio) was applied. Images were acquired using a fluorescence microscope (Nikon, Japan). For the positive control, permeabilized sections were treated with DNase I (G3342, Servicebio) before staining, whereas for the negative control, sections were incubated with TdT buffer without recombinant TdT enzyme. Three samples from each group were randomly selected for quantification of the apoptosis rate (TUNEL-positive cells/total nuclei), which was performed by a blinded investigator using ImageJ software.

### Biochemical index detection

Mouse serum was collected and analyzed using commercial kits (Jiangsu Meimian, Nanjing Jiancheng) to determine AD-related core biomarkers, including Aβ1-40 (MM-0461M1), Aβ1-42 (MM-0220M1), TAU181 (MM-46827M1), and γ-secretase (MM-44563M1). OS-related indicators were also measured, including SOD (A001-3), CAT (A007-1-1), GSH (A006-2-1), GSH-PX (A005-1-2), MDA (A003-1-1), GSH (A006-2-1), and GSSG (A061-1-2). Absorbance was read using a multifunctional microplate reader (K6600B, Keiao, China).

### Western blot (WB)

WB was used to assess p-JNK/JNK, p-ERK/ERK, and p-p38/p38 expression. Tissue proteins were extracted with RIPA buffer (89901, Thermo Fisher Scientific, USA) containing protease and phosphatase inhibitors (PPC1010, Sigma-Aldrich, USA), and quantified using a BCA kit (23225, Thermo Fisher Scientific, USA). Equal protein amounts were resolved by SDS-PAGE, transferred to PVDF membranes (88518, Thermo Fisher Scientific, USA), and blocked with 5 % BSA for 1.5 h. Membranes were incubated overnight at 4 °C with primary antibodies against p38 MAPK (#8690S), phosphorylated p38 MAPK (#4511S), ERK (#9102S), phosphorylated ERK (#9101S), JNK (#9252S), phosphorylated JNK (#9251S), and β-actin (3700), all at 1:1,000 (Cell Signaling Technology, USA). After washing, membranes were incubated with HRP-conjugated secondary antibodies (ab6721, 1:5,000, Abcam) and developed using ECL reagents (BL520A, Biosharp). Images were obtained with a Bio-Rad imaging system, and band intensities were analyzed using ImageJ, with results expressed as target protein/β-actin ratios.

### Statistical analysis

All statistical analyses were performed using SPSS 17.0 software (Media Cybernetics, Inc., Rockville, MD, USA). Data are presented as mean ± standard error of the mean (SEM). Group comparisons were performed by one-way ANOVA with Bonferroni post hoc tests, and normality was verified for small-sample datasets before ANOVA. Differences were considered significant at p<0.05.

## Results

### Effects of ESP treatment on body weight in APP/PS1 mice

**Figure 2:**
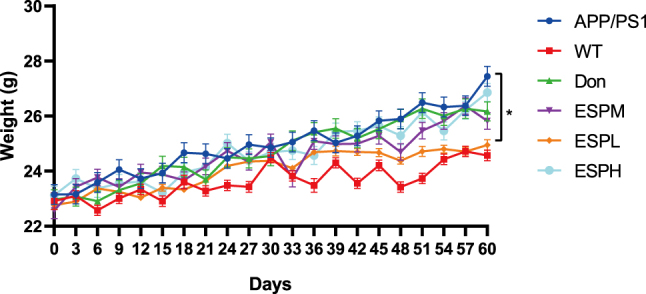
Changes in body weight of mice. Note: body weight changes in mice from each experimental group during the treatment period. Each group contained 10 mice, and body weight is expressed as mean±SEM. Statistical analysis was performed using one-way ANOVA, followed by Fisher’s LSD t-test for multiple comparisons.

During the 60-day treatment, no mortality or severe adverse events were observed. Body weight increased progressively in all groups. Compared with WT mice, APP/PS1 model mice showed excessive body-weight gain and exhibited the highest body weight by day 60 (Figure 2). Drug intervention attenuated this excessive increase, as body weights in the Don and ESP-treated groups were lower than those in APP/PS1 model mice. Baseline body weight at day 0 showed no significant difference among groups, indicating balanced weight distribution before treatment. No signs of alopecia, gastrointestinal abnormalities, hunching, lethargy, or other toxicity-related phenotypes were observed in any treatment group.

### ESP improves cognitive function in APP/PS1 mice

**Figure 3:**
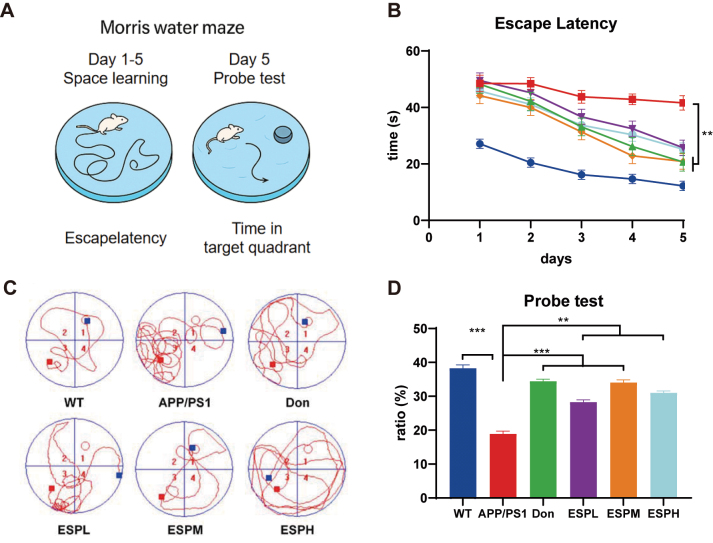
ESP treatment alleviates learning and memory deficits in APP/PS1 mice. Note: (A) schematic diagram of the MWM test; (B) escape latency of each group during the MWM training period (days 1–5); (C) swimming trajectory plots from the probe trial on day 5, where the red square indicates the starting position, the blue mark indicates the ending position, and the red circle denotes the target platform; (D) percentage of time spent in the target quadrant by each group during the probe trial. Statistical analysis was performed using one-way ANOVA, followed by Bonferroni multiple comparison tests. ***p<0.001, **p<0.01, *p<0.05. Data are presented as mean ± SEM (n=10).

In the MWM test (Figure 3A), APP/PS1 mice showed significantly longer escape latencies than WT controls (p<0.001), reflecting impaired spatial learning and memory. Following ESP intervention, cognitive performance in APP/PS1 mice improved to varying degrees. All ESP-treated groups showed significantly reduced escape latencies compared with the model group, with the greatest improvement in the ESPM (200 mg/kg) group (p<0.01 vs. APP/PS1). On day 5, no significant difference in escape latency was detected between the ESPM group and the positive control group treated with Don (0.5 mg/kg) (p>0.05), suggesting that the cognitive improvement in the ESPM group was comparable to that of Don (Figure 3B).

In the probe test, WT mice spent 38.26% of the time in the target quadrant, indicating strong spatial memory, whereas APP/PS1 model mice showed a significant reduction (p<0.001 vs. WT). ESP treatment improved memory retention, with the ESPL and ESPH groups spending more time in the target quadrant than the model group (p<0.01). The ESPM group performed comparably to the Don group, with no significant difference between them (p>0.05) (Figures 3C and D).

These findings indicate that ESP treatment effectively alleviated learning and memory deficits in APP/PS1 mice, with the medium dose producing an intervention effect comparable to that of the standard drug.

### Effects of ESP treatment on hippocampal pathology and neurons in APP/PS1 mice

**Figure 4:**
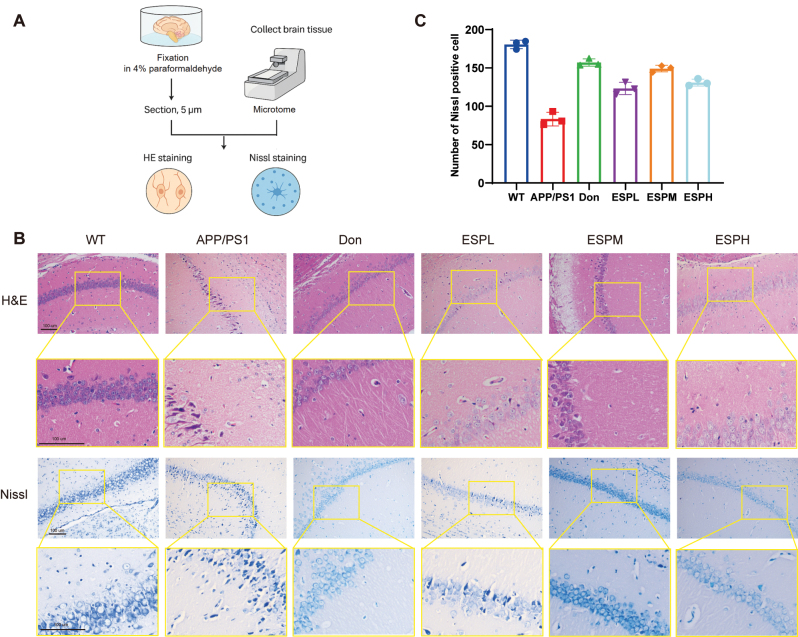
Effects of ESP on the hippocampal CA1 region. Note: (A) representative images of H&E and Nissl staining; (B) H&E and Nissl staining of the hippocampal CA1 region, scale bar=100 μm; (C) quantitative analysis of Nissl-stained CA1 region, showing the number of positively stained cells. n=3; data are presented as mean ± SEM; ***p<0.001 vs. WT group; ^###^p<0.001 vs. APP/PS1 group.

To assess hippocampal neuronal pathology in APP/PS1 mice, H&E and Nissl staining were performed on tissue sections from each group (Figure 4A).

H&E staining revealed that WT mice exhibited well-defined hippocampal architecture, with neurons arranged in a regular, dense pattern, intact cell bodies, complete nuclear membranes, and no evidence of pyknosis or other pathological changes. In contrast, hippocampal tissues from APP/PS1 mice showed numerous neurons with chromatin condensation, nuclear pyknosis, and disrupted structural organization, indicating severe neuronal injury. ESP treatment alleviated these structural abnormalities to varying degrees. Notably, the ESPM group displayed relatively orderly neuronal arrangement, well-preserved nuclear structure, and markedly reduced pathological changes, closely resembling the Don group. The ESPH and ESPL groups also exhibited some degree of structural improvement, though the effect was less pronounced (Figure 4B).

Nissl staining further supported these findings. APP/PS1 mice had a marked reduction and disorganized distribution of Nissl bodies, whereas ESP-treated groups – particularly the ESPM group – showed increased numbers and more concentrated distribution of Nissl bodies. These results suggest that ESP improved neuronal survival and exerted neuroprotective effects (Figures 4B and C).

### ESP attenuates neuronal apoptosis in the hippocampus

**Figure 5:**
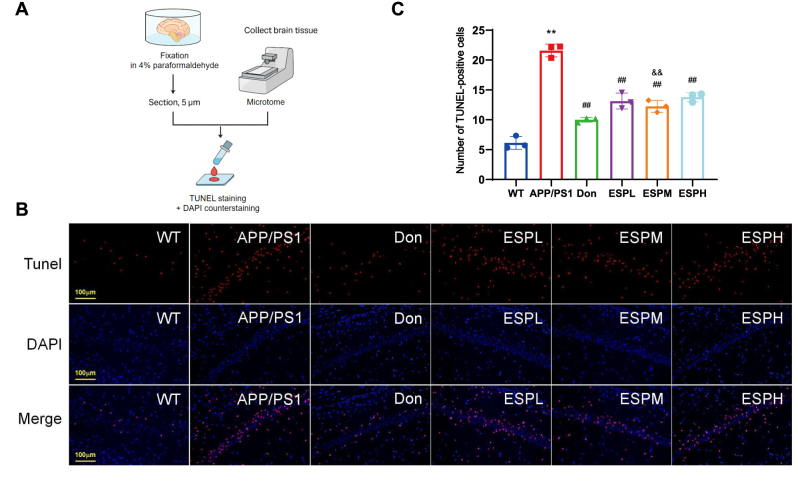
ESP treatment reduces neuronal apoptosis in the hippocampus of APP/PS1 mice. Note: (A) representative schematic of TUNEL staining; (B) representative TUNEL-stained images showing apoptosis in the hippocampal region; (C) quantitative analysis of TUNEL-positive cells, illustrating the effect of ESP on hippocampal neuronal apoptosis. Scale bar=100 μm **p<0.01 vs. WT group; ^##^p<0.01 vs. APP/PS1 group; and ^&&^p<0.01 vs. Don group. Data are presented as mean±SEM (n=3).

To further assess neuronal survival, hippocampal apoptosis in APP/PS1 mice was evaluated by TUNEL staining (Figure 5A). As shown in the TUNEL staining results (Figure 5B), WT mice exhibited minimal red fluorescence signals in the hippocampus, with intact structure and orderly cell arrangement, indicating normal neuronal viability. In contrast, the APP/PS1 model group displayed markedly increased red fluorescence signals and disorganized neuronal arrangement, suggesting extensive apoptosis. ESP treatment markedly reduced TUNEL-positive signals in the hippocampus, particularly in the ESPM group (200 mg/kg), where neuronal arrangement appeared more orderly, the number of apoptotic cells was greatly reduced, and structural integrity was better preserved. Similar improvements were also observed in the Don group.

Quantitative analysis of TUNEL-positive cells (Figure 5C) further confirmed these findings. The APP/PS1 group showed a significantly higher apoptosis rate than WT mice (p<0.01), whereas all ESP-treated groups exhibited marked reductions compared with the model group (p<0.01). Apoptosis in the ESPM group was close to that of the Don group, although a significant difference remained (p<0.01), indicating a strong but not fully equivalent neuroprotective effect.

In summary, ESP intervention effectively inhibited hippocampal neuronal apoptosis in APP/PS1 mice, showing a degree of dose dependence, with the medium dose producing the most pronounced effect.

### ESP reduces circulating AD-associated biomarkers in APP/PS1 mice

**Figure 6:**
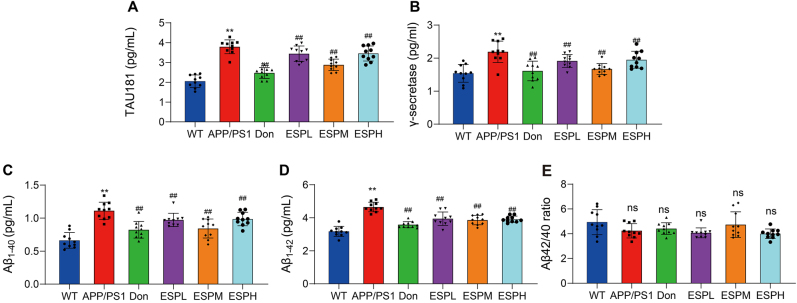
ESP treatment improves core AD biomarkers in APP/PS1 mice. Note: serum levels of core AD biomarkers in each group: (A) TAU181; (B) γ-secretase; (C) Aβ1-40; (D) Aβ1-42; (E) Aβ1-42/Aβ1-40 ratio. **p<0.01 vs. WT group; ^##^p<0.01 vs. APP/PS1 group. Data are presented as mean ± SEM (n=10).

Serum levels of TAU181, γ-secretase, Aβ1-40, and Aβ1-42 were determined by ELISA. Compared with WT mice, all four markers were significantly increased in APP/PS1 mice (p<0.01), confirming the presence of AD-like pathology (Figure 6A–D). Additionally, the Aβ1-42/Aβ1-40 ratio was markedly reduced in the APP/PS1 group (Figure 6E), reflecting abnormal Aβ metabolism and pathological cleavage imbalance. ESP treatment significantly decreased all four biomarkers, with the strongest effects in the ESPM group, where TAU181, γ-secretase, Aβ1-40, and Aβ1-42 levels were markedly reduced compared with the APP/PS1 group (p<0.01). In addition, the Aβ1-42/Aβ1-40 ratio in the ESPM group was restored toward WT levels, indicating that ESP may improve Aβ metabolism by limiting both Aβ accumulation and its toxic species.

In summary, ESP treatment effectively mitigated the abnormal expression of core serum biomarkers in AD model mice, demonstrating dose dependence and potential disease-modifying effects.

### ESP treatment attenuates oxidative damage in APP/PS1 mice

**Figure 7:**
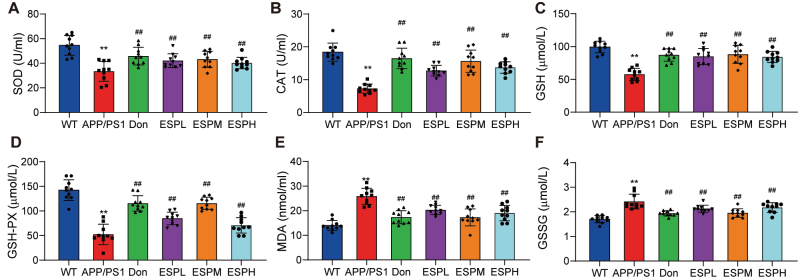
ESP treatment alleviates oxidative damage in APP/PS1 mice. Note: serum levels of antioxidant enzymes and oxidative damage markers in each group: (A) SOD; (B) CAT; (C) GSH; (D) GSH-PX; (E) MDA; (F) GSSG. **p<0.01 vs. WT group; ^##^p<0.01 vs. APP/PS1 group. Data are presented as mean ± SEM (n=10).

To assess the effect of ESP on oxidative stress, serum levels of SOD, CAT, GSH, and GSH-PX, together with MDA and GSSG, were measured in APP/PS1 mice (Figure 7A–F). Compared with the WT group, APP/PS1 mice exhibited significantly reduced levels of SOD, CAT, GSH, and GSH-PX (Figure 7A–D), indicating impaired antioxidant defense capacity and diminished ability to eliminate ROS. ESP significantly elevated antioxidant indices, with the greatest recovery in the ESPM group, where several parameters approached WT levels, indicating enhanced antioxidant capacity.

In addition, serum levels of MDA and GSSG were markedly elevated in APP/PS1 mice (Figure 7E and F), reflecting increased lipid peroxidation and OS. ESP treatment markedly reduced MDA and GSSG concentrations, with the most notable improvement observed in the ESPM group (p<0.01), indicating that ESP intervention helps mitigate OS-related cellular damage.

In summary, ESP effectively alleviated OS in AD model mice by enhancing the antioxidant defense system, reducing free radical accumulation, and decreasing lipid peroxidation.

### ESP treatment inhibits MAPK signaling pathway in APP/PS1 mice

**Figure 8:**
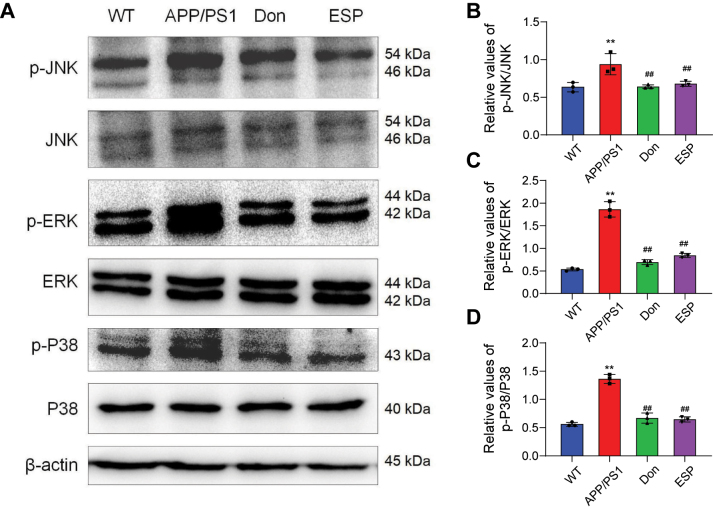
ESP (200 mg/kg) treatment inhibits MAPK signaling pathway in APP/PS1 mice. Note: (A) representative western blot bands of p-JNK, JNK, p-ERK, ERK, p-p38, and p38 proteins in the hippocampus of each group; (B) quantitative analysis of p-JNK/JNK; (C) quantitative analysis of p-ERK/ERK; (D) quantitative analysis of p-p38/p38. **p<0.01 vs. WT group; ^##^p<0.01 vs. APP/PS1 group. Data are presented as mean ± SEM (n=3).

Previous studies indicate that MAPK signaling is critically involved in AD development, especially in regulating Aβ generation and pathological Tau phosphorylation [27], [28], [29]. To further determine whether ESP exerts neuroprotective effects through regulation of the MAPK pathway, we examined the hippocampal levels of JNK, ERK, and p38, along with their phosphorylated forms, in APP/PS1 mice (Figure 8A–D).

WB showed that hippocampal p-JNK, p-ERK, and p-p38 levels were markedly elevated in APP/PS1 mice compared with WT controls (p<0.01), indicating strong MAPK pathway activation. ESP treatment significantly suppressed this activation, with the ESPM group (200 mg/kg) showing the greatest reductions in p-JNK, p-ERK, and p-p38 relative to the model group (all p<0.01), suggesting that ESP effectively suppressed excessive MAPK pathway activation in the hippocampus of AD model mice (Figure 8A–D).

In summary, ESP may alleviate pathological changes in APP/PS1 mice by inhibiting abnormal activation of the MAPK signaling pathway, thereby exerting neuroprotective effects.

## Discussion

AD is a progressive neurodegenerative condition linked to aging and characterized by cognitive decline, neuronal loss, and structural brain deterioration [30]. While the pathogenesis of AD remains incompletely elucidated, numerous studies implicate a range of mechanisms, including Aβ accumulation, disrupted calcium signaling, impaired autophagy, apoptosis, and chronic neuroinflammation [31], [32], [33]. OS, in particular, has emerged as a central factor in neuronal vulnerability and disease progression, given the high metabolic demand and limited antioxidant defenses of neurons [29], 34], 35].

During the experiment, ESP exhibited a dose-dependent effect on body weight regulation. Although ESPM mice started with slightly lower weights, their gain was moderate and ultimately stabilized near WT levels, while remaining significantly lower than in the Don group (p<0.05). This trend suggests that ESP, at certain doses, may modulate energy metabolism or influence gastrointestinal function, thereby indirectly affecting body weight. The potential mechanisms may include: first, ESP may contain bioactive components that enhance mitochondrial function, promote fatty acid oxidation or glucose metabolism, and thereby increase basal metabolic rate; second, ESP may regulate gastrointestinal motility or alter the gut microbiota composition, influencing nutrient absorption efficiency. These observations are consistent with traditional Tibetan medicine records describing certain components as capable of regulating “stomach fire” and improving digestive function [36]. Notably, no obvious toxic reactions were observed in any experimental group, indicating that the observed body weight changes are more likely attributable to the metabolic effects of ESP rather than malnutrition or toxic side effects. Furthermore, unexplained weight loss is a well-documented systemic manifestation of AD and has been shown to correlate with disease progression and cognitive decline in both clinical cohorts and preclinical studies [37], [38], [39]. Emerging evidence also indicates that systemic metabolic dysregulation, including impaired energy homeostasis and nutritional imbalance, can exacerbate neuronal energy deficits and accelerate neurodegenerative pathology [40]. Therefore, the ability of ESP to stabilize body weight may reflect an improvement in overall metabolic health. By preventing pathological weight loss, ESP could provide better systemic energetic support for the brain, thereby indirectly contributing to the preservation of cognitive function. Although the median lethal dose (LD_50_) of ESP was not determined in the present study, its safety profile is supported by existing pharmacopoeial standards and prior preclinical and clinical evidence. ESP is an officially approved Tibetan medicine formulation listed in the Chinese Pharmacopoeia (2020 edition), indicating that it has undergone mandatory safety and toxicity evaluations required for clinical use. Previous experimental studies have shown that repeated oral administration of ESP did not induce overt toxicity or significant histopathological alterations in major organs, including the liver and kidneys [22]. In addition, clinical investigations in epilepsy patients demonstrated favorable safety outcomes and good tolerability during prolonged ESP treatment, with no severe adverse reactions reported [21]. Furthermore, experimental studies employing ESP in neurological disease models have consistently reported the absence of observable systemic toxicity at therapeutic doses [41]. Importantly, the individual constituents of ESP have also been shown to exhibit low acute toxicity. For example, saffron-derived crocin and related compounds display a wide safety margin in preclinical studies [42], 43], while mineral components such as coral and pearl are classified as low-toxicity materials in traditional pharmacopoeias. Although ESP contains cinnabar (HgS), its content is strictly regulated, and HgS is characterized by extremely low gastrointestinal bioavailability, substantially limiting systemic mercury exposure. Collectively, these data indicate that the highest dose used in this study (400 mg/kg) remains well below reported toxic thresholds and within a safe therapeutic window.

In the present study, ESP demonstrated robust neuroprotective effects in APP/PS1 mice, evidenced by improved cognitive performance, reduced hippocampal apoptosis, and preserved neuronal morphology. ESP administration significantly enhanced the activity of antioxidant enzymes and suppressed OS markers, suggesting that redox regulation is a key mechanism underlying its effects. Additionally, ESP inhibited phosphorylation of key MAPK pathway components (ERK, JNK, and p38), implicating MAPK signaling modulation as another therapeutic target. These findings support the notion that ESP exerts its benefits via a multi-target approach – addressing oxidative imbalance, apoptosis, and aberrant signaling cascades simultaneously. This aligns with emerging therapeutic paradigms that emphasize multi-modal strategies over single-target interventions in complex disorders like AD [44], 45]. Overall, these findings indicate that ESP is a promising multi-target candidate with translational potential for AD therapy. The selection of a 60 day treatment duration in this study was critical. While ESP has been historically utilized for chronic neurological disorders such as epilepsy and stroke recovery, specific controlled studies on its long-term effects in AD models have been lacking. Our findings confirm that this extended regimen effectively covers the critical window of pathological progression in APP/PS1 mice, allowing sufficient time for the multi-target mechanisms of ESP – antioxidation and signaling modulation – to manifest structural and functional benefits.

Don was approved by the China Food and Drug Administration in 2006 for the treatment of mild to moderate AD and in 2017 for the treatment of severe AD [46]. Compared with the conventional AD therapeutic agent Don, ESP not only demonstrated efficacy in improving cognitive function but also exhibited pronounced neuroprotective effects. In this study, the therapeutic efficacy of ESPM (200 mg/kg) was comparable to that of the Don group (0.5 mg/kg) in alleviating learning deficits, preserving hippocampal structure, improving serum AD core biomarker levels, and ameliorating OS parameters, consistent with the findings of Burton BR et al. (2014) [47]. ESP treatment markedly reduced pathological neuronal damage in the hippocampus, indicating its potential to preserve neuronal morphology. These findings provide strong support for the development of ESP as a novel therapeutic agent for AD.

**Figure 9:**
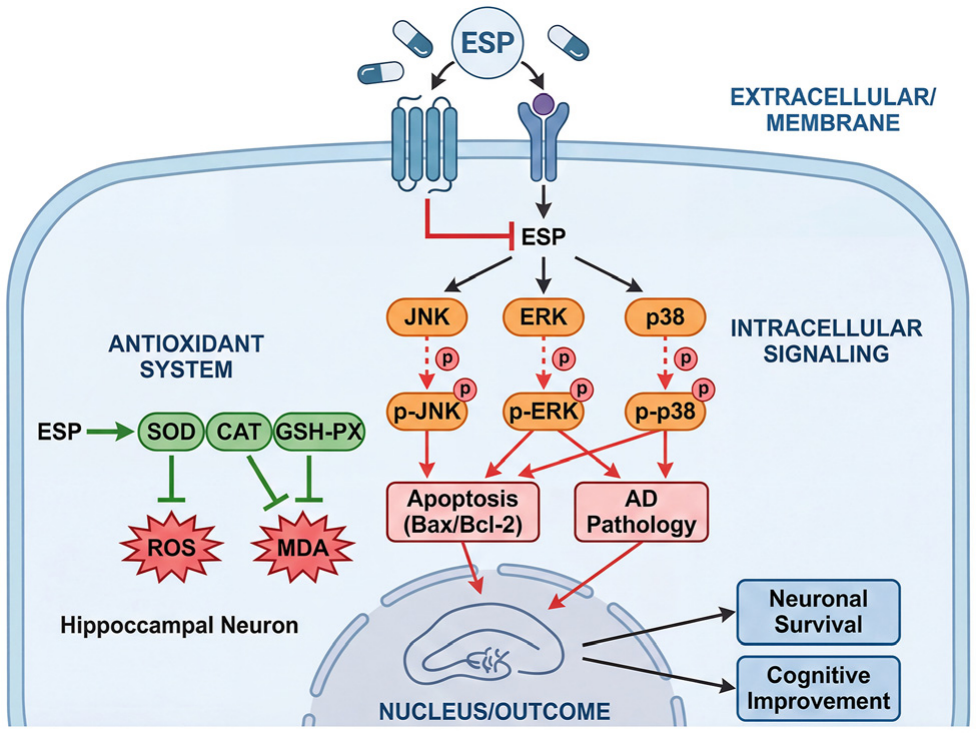
Schematic representation of the potential neuroprotective mechanisms of ESP in APP/PS1 mice. Note: proposed molecular mechanism of ESP in hippocampal neurons. Left: ESP enhances antioxidant defenses (SOD, CAT, GSH-PX) to scavenge ROS and reduce MDA. Right: ESP inhibits the phosphorylation of MAPK pathway components (JNK, ERK, p38), thereby blocking downstream apoptotic signaling and AD pathology. Together, these actions promote neuronal survival and cognitive improvement. Green arrows: activation/promotion; red T-bars: inhibition; red arrows: Signaling flow/pathological progression; p: phosphorylation.

TUNEL staining analysis revealed that ESP significantly reduced the neuronal apoptosis rate in the hippocampus of APP/PS1 mice, consistent with recent studies emphasizing the pivotal role of neuroprotection in AD therapy [7], 48]. The strong anti-apoptotic effect of ESP further supports its neuroprotective potential in AD. Together with the overall findings, these results suggest that ESP acts through multiple mechanisms, providing new insights into its clinical applicability. Consistent with previous reports, ESP markedly reduced the phosphorylation of JNK, ERK, and p38 MAPK, in line with the findings of Wang et al. [24]. Given the established role of MAPK signaling in AD pathogenesis [19], 20], inhibition of this pathway by ESP further supports its therapeutic promise. Based on these results, we propose that ESP confers neuroprotection by enhancing antioxidant defenses to reduce ROS while simultaneously inhibiting JNK, ERK, and p38 phosphorylation in the MAPK pathway to limit apoptosis (Figure 9).

Despite these encouraging results, several limitations should be acknowledged. First, the study relied exclusively on the APP/PS1 transgenic mouse model, which, although widely adopted and representative of core AD features such as Aβ deposition and cognitive decline [49], does not fully capture the complexity of human AD pathology. Notably, astrocyte biology differs significantly between species. For instance, Zhou et al. [50] reported that while murine astrocytes exhibit modest Aβ-induced GFAP activation, human AD brains show impaired astrocytic subpopulations involved in lipid metabolism and detoxification, suggesting species-specific glial dysfunction. Furthermore, only male mice were used, leaving potential sex-dependent treatment effects unexplored. Behavioral evaluation was limited to the MWM, which – despite its established role in assessing spatial learning – may not adequately reflect other cognitive domains. Additional behavioral assays, such as the Y-maze, novel object recognition, and fear conditioning, would provide a more comprehensive assessment. In addition, inflammatory mediators such as IL-1β and TNF-α were not assessed, restricting insight into ESP’s potential anti-inflammatory actions. Biomarker analysis was confined to serum samples, with no parallel investigation of brain tissue levels, limiting our interpretation of direct neurobiological effects.

While ESP significantly improved multiple pathological and behavioral outcomes, no clear dose-response trend was observed. The ESPM (200 mg/kg) consistently outperformed both ESPL and ESPH. This non-linear dose-response relationship, often referred to as “hormesis,” is frequently observed in traditional multi-herb formulations. Several factors may contribute to the diminished efficacy at the high dose (400 mg/kg). First, the absorption of bioactive components in ESP may depend on specific transporters that become saturated at higher concentrations, leading to a plateau or reduction in bioavailability. Second, as a complex mixture, ESP contains numerous compounds; while some exert neuroprotective effects, others present at higher concentrations might trigger counter-regulatory mechanisms or mild toxicity that offsets therapeutic benefits. For instance, excessive activation of certain signaling pathways or metabolic burden on the liver and kidneys to eliminate high concentrations of xenobiotics could dampen the overall neuroprotective outcome. This “inverted U-shaped” efficacy curve underscores the importance of precise dosage optimization in clinical applications of Tibetan medicine. This highlights the need for future studies examining ESP’s bioavailability and metabolism. Moving forward, translational research should incorporate rigorous pharmacokinetic and pharmacodynamic analyses to define optimal dosing regimens; sex- and age-balanced experimental designs to evaluate differential biological responses; expanded behavioral and cognitive phenotyping; and comprehensive inflammatory and molecular profiling in both serum and brain tissue. In parallel, well-designed clinical trials will be required to establish safety and efficacy in human populations. Collectively, these efforts will be essential for validating ESP as a multi-target therapeutic agent and for advancing its clinical development in AD.

## Conclusions

This study demonstrates that ESP exert therapeutic effects in APP/PS1 transgenic mice through multiple mechanisms. ESP treatment significantly improved cognitive performance, reduced hippocampal neuronal apoptosis, preserved cellular morphology, lowered serum levels of Aβ1-40, Aβ1-42, TAU181, and γ-secretase, enhanced antioxidant enzyme activities, and suppressed MAPK pathway activation (Graphical Abstract). These findings provide experimental evidence supporting the significance of ESP as a multi-target therapeutic agent for AD. By simultaneously modulating OS, apoptosis, and key signaling pathways, ESP offers a promising alternative to conventional single-target therapies and expands the molecular framework for AD intervention strategies.

Nevertheless, as this study was limited to a murine model, the clinical relevance of ESP remains to be validated. Future studies should clarify the underlying mechanisms of ESP, refine dosing regimens, and assess its safety and efficacy in clinical settings. Comprehensive mechanistic and translational studies will be essential to advance ESP toward therapeutic application in AD and inform novel prevention and treatment modalities.
